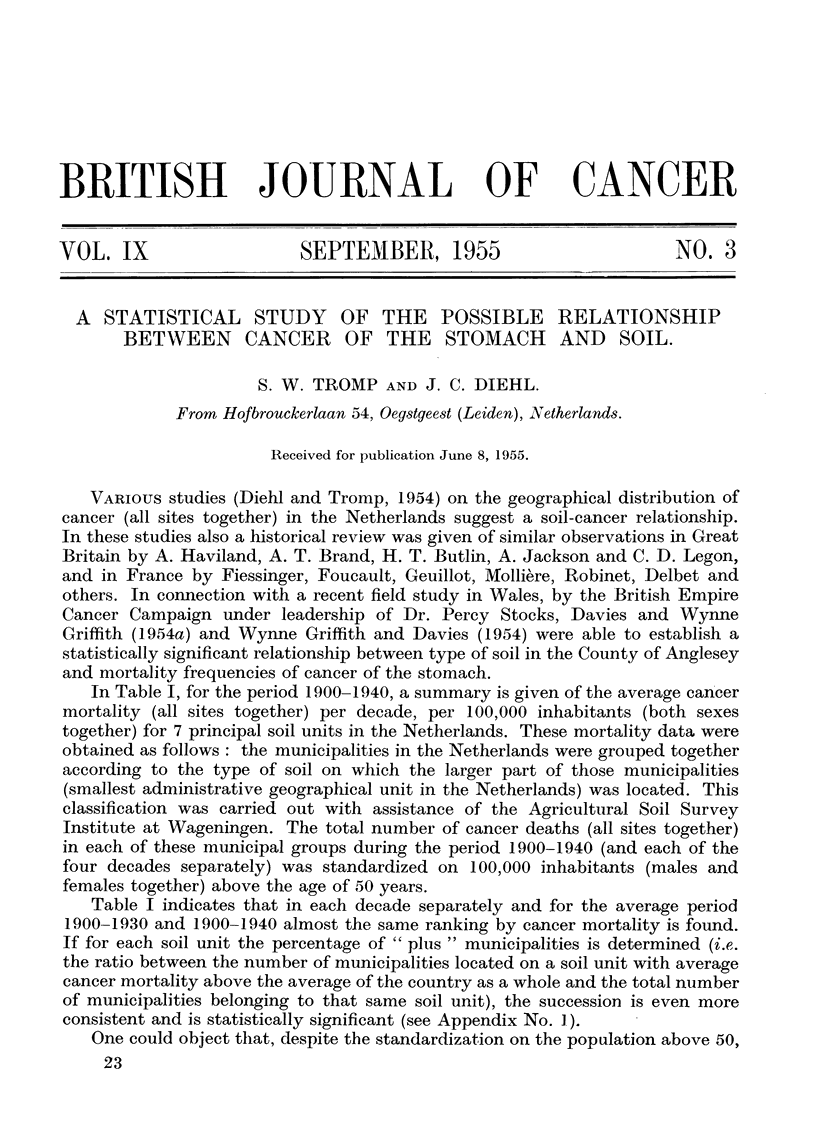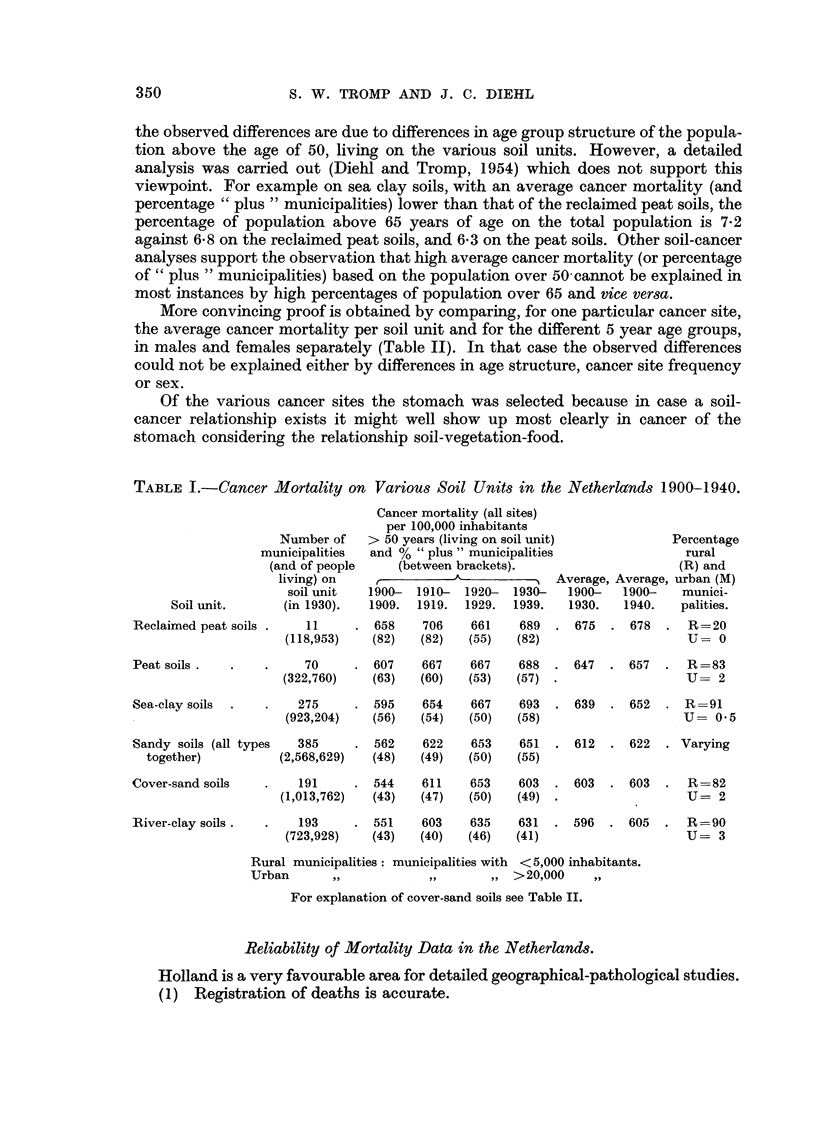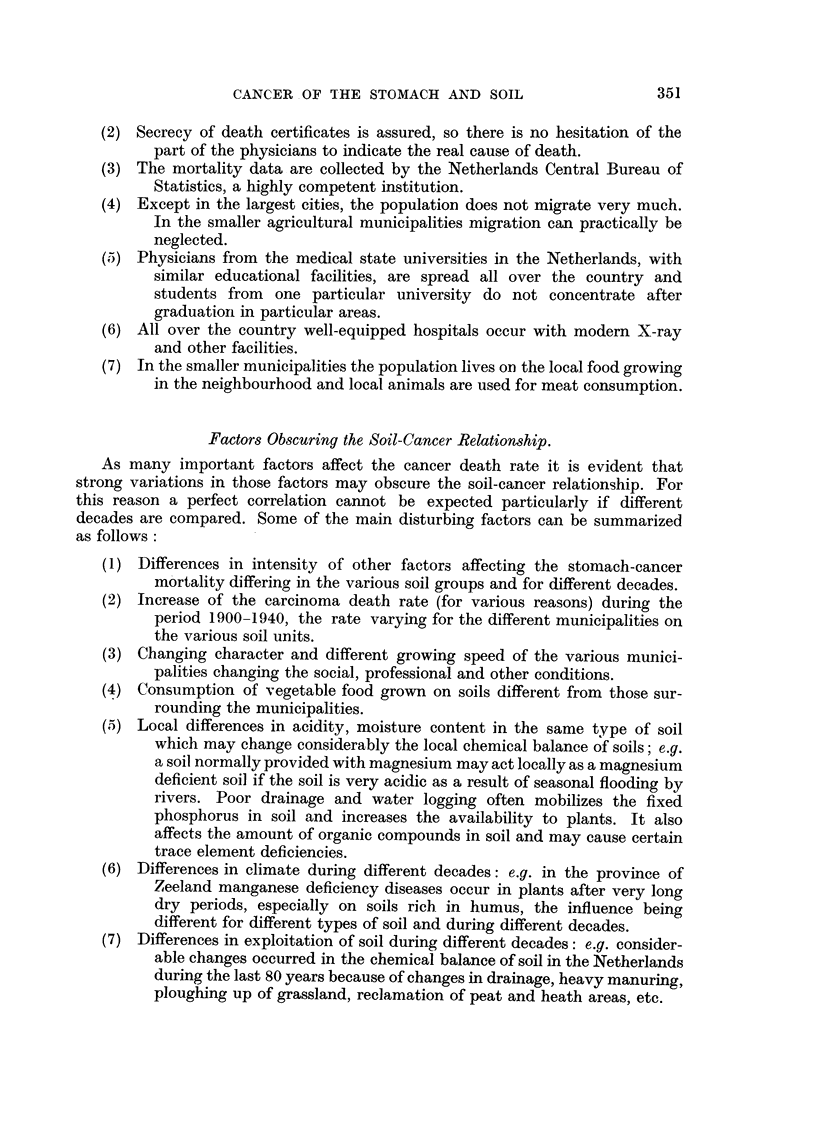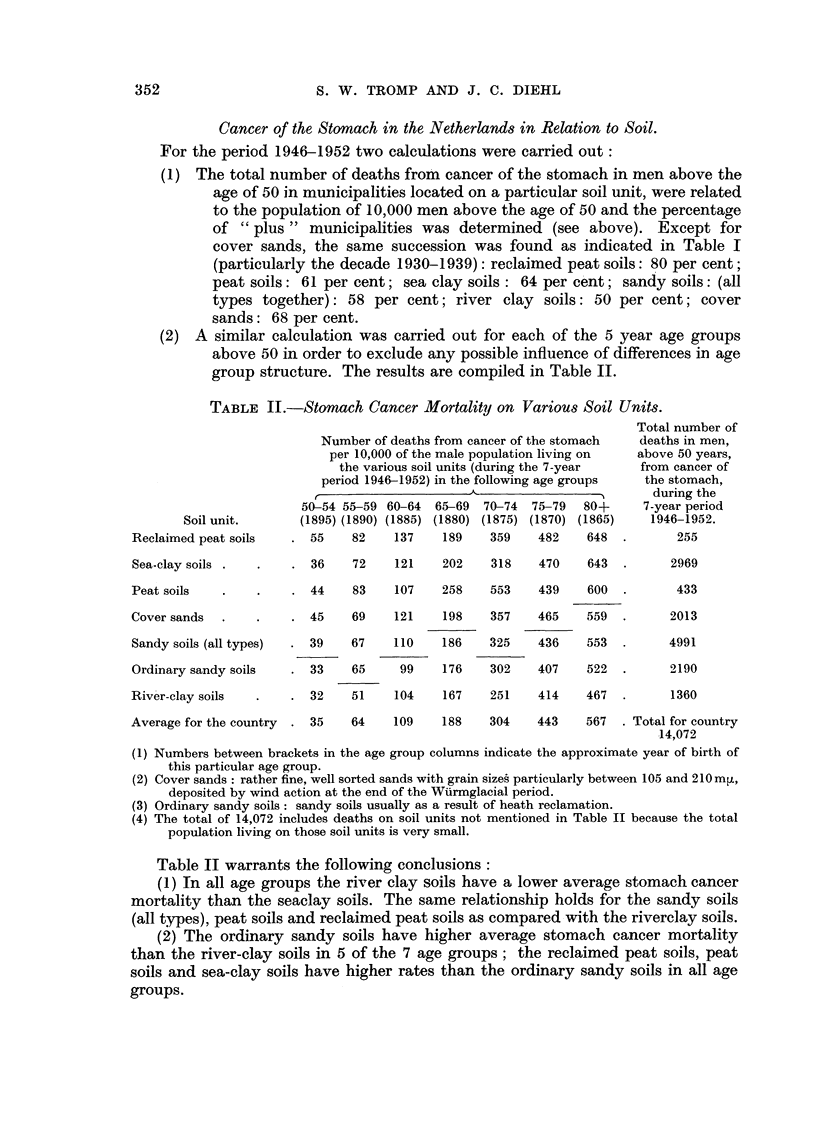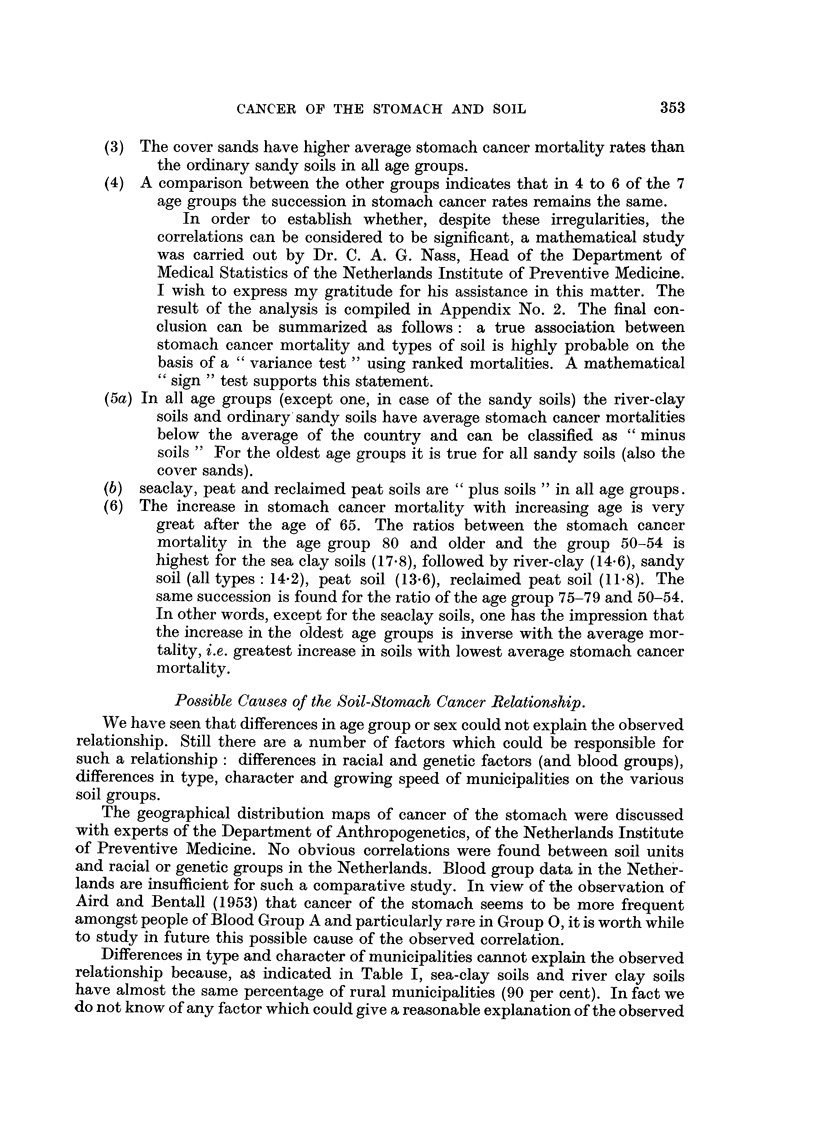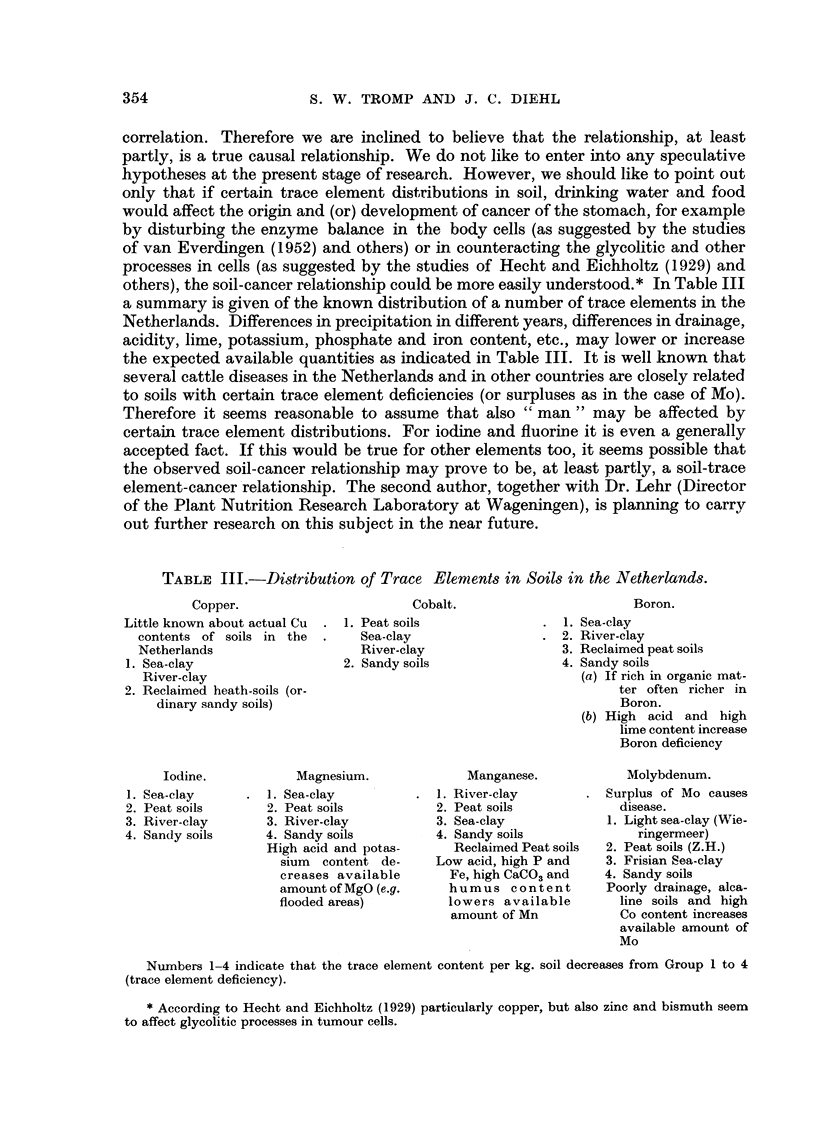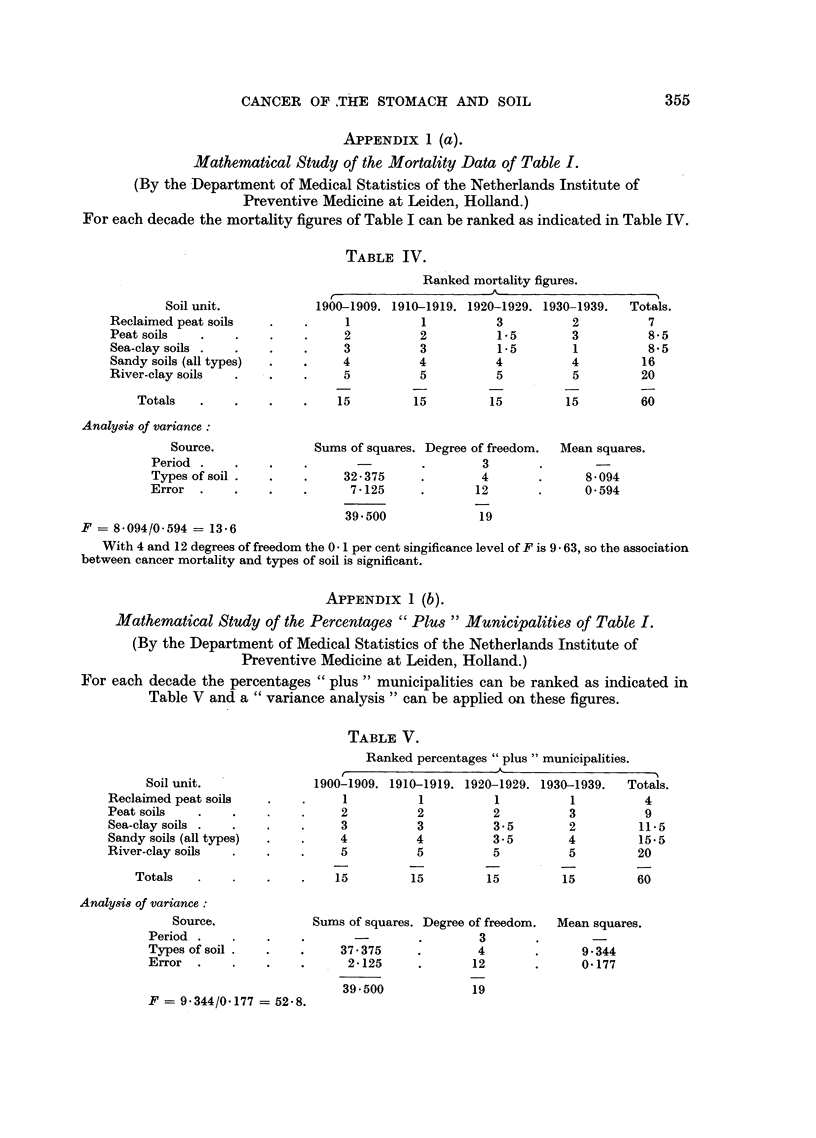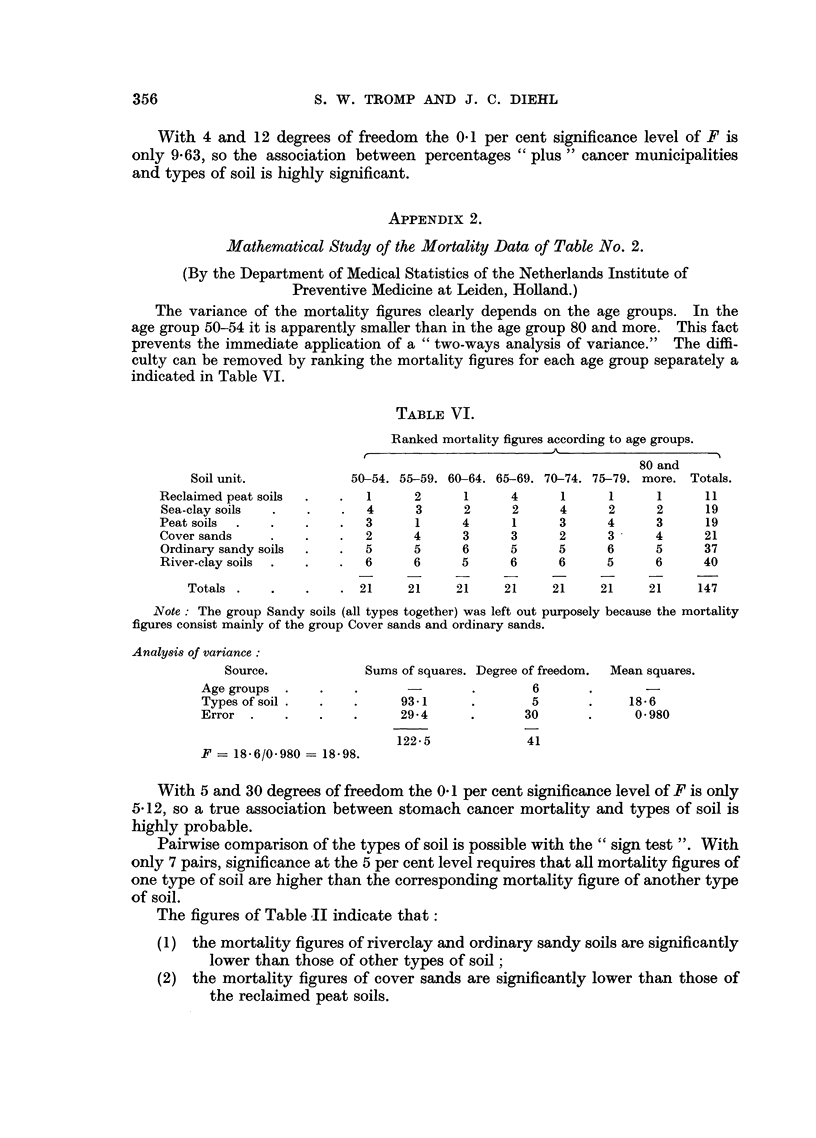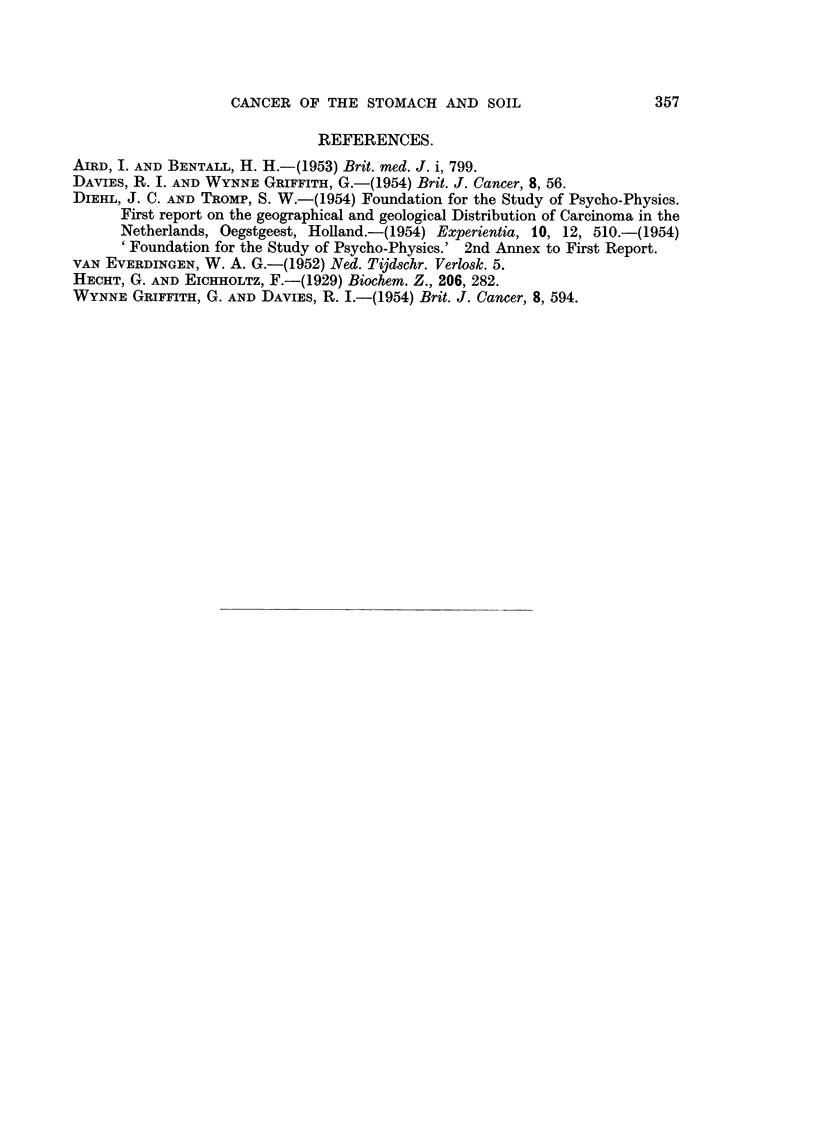# A Statistical Study of the Possible Relationship between Cancer of the Stomach and Soil

**DOI:** 10.1038/bjc.1955.33

**Published:** 1955-09

**Authors:** S. W. Tromp, J. C. Diehl


BRITISH JOURNAL OF CANCER

VOL. IX SEPTEMBER, 1955 NO. 3

### A STATISTICAL STUDY OF THE POSSIBLE RELATIONSHIP BETWEEN CANCER OF THE STOMACH AND SOIL.

S. W. TROMP AND J. C. DIEHL.

*From Hofbrouckerlaan 54, Oegstgeest (Leiden), Netherlands.*

Received for publication June 8, 1955.

VARIOUS studies (Diehl and Tromp, 1954) on the geographical distribution of cancer (all sites together) in the Netherlands suggest a soil-cancer relationship. In these studies also a historical review was given of similar observations in Great Britain by A. Haviland, A. T. Brand, H. T. Butlin, A. Jackson and C. D. Legon, and in France by Fiessinger, Foucault, Geuillot, Mollière, Robinet, Delbet and others. In connection with a recent field study in Wales, by the British Empire Cancer Campaign under leadership of Dr. Percy Stocks, Davies and Wynne Griffith (1954*a*) and Wynne Griffith and Davies (1954) were able to establish a statistically significant relationship between type of soil in the County of Anglesey and mortality frequencies of cancer of the stomach.

In Table I, for the period 1900–1940, a summary is given of the average cancer mortality (all sites together) per decade, per 100,000 inhabitants (both sexes together) for 7 principal soil units in the Netherlands. These mortality data were obtained as follows : the municipalities in the Netherlands were grouped together according to the type of soil on which the larger part of those municipalities (smallest administrative geographical unit in the Netherlands) was located. This classification was carried out with assistance of the Agricultural Soil Survey Institute at Wageningen. The total number of cancer deaths (all sites together) in each of these municipal groups during the period 1900–1940 (and each of the four decades separately) was standardized on 100,000 inhabitants (males and females together) above the age of 50 years.

Table I indicates that in each decade separately and for the average period 1900–1930 and 1900–1940 almost the same ranking by cancer mortality is found. If for each soil unit the percentage of " plus " municipalities is determined (*i.e.* the ratio between the number of municipalities located on a soil unit with average cancer mortality above the average of the country as a whole and the total number of municipalities belonging to that same soil unit), the succession is even more consistent and is statistically significant (see Appendix No. 1).

One could object that, despite the standardization on the population above 50, the observed differences are due to differences in age group structure of the population above the age of 50, living on the various soil units. However, a detailed analysis was carried out (Diehl and Tromp, 1954) which does not support this viewpoint. For example on sea clay soils, with an average cancer mortality (and percentage " plus " municipalities) lower than that of the reclaimed peat soils, the percentage of population above 65 years of age on the total population is 7·2 against 6·8 on the reclaimed peat soils, and 6·3 on the peat soils. Other soil-cancer analyses support the observation that high average cancer mortality (or percentage of " plus " municipalities) based on the population over 50 cannot be explained in most instances by high percentages of population over 65 and *vice versa.*

More convincing proof is obtained by comparing, for one particular cancer site, the average cancer mortality per soil unit and for the different 5 year age groups, in males and females separately (Table II). In that case the observed differences could not be explained either by differences in age structure, cancer site frequency or sex.

Of the various cancer sites the stomach was selected because in case a soil-cancer relationship exists it might well show up most clearly in cancer of the stomach considering the relationship soil-vegetation-food.

TABLE I.—*Cancer Mortality on Various Soil Units in the Netherlands* 1900–1940.

| Soil unit. | Number of municipalities (and of people living) on soil unit (in 1930). | Cancer mortality (all sites) per 100,000 inhabitants > 50 years (living on soil unit) and % " plus " municipalities (between brackets). | | | | Average, 1900–1930. | Average, 1900–1940. | Percentage rural (R) and urban (M) municipalities. |
|---|---|---|---|---|---|---|---|---|
| | | 1900–1909. | 1910–1919. | 1920–1929. | 1930–1939. | | | |
| Reclaimed peat soils | 11 | 658 | 706 | 661 | 689 | 675 | 678 | R=20 |
| | (118,953) | (82) | (82) | (55) | (82) | | | U= 0 |
| Peat soils | 70 | 607 | 667 | 667 | 688 | 647 | 657 | R=83 |
| | (322,760) | (63) | (60) | (53) | (57) | | | U= 2 |
| Sea-clay soils | 275 | 595 | 654 | 667 | 693 | 639 | 652 | R=91 |
| | (923,204) | (56) | (54) | (50) | (58) | | | U= 0·5 |
| Sandy soils (all types together) | 385 | 562 | 622 | 653 | 651 | 612 | 622 | Varying |
| | (2,568,629) | (48) | (49) | (50) | (55) | | | |
| Cover-sand soils | 191 | 544 | 611 | 653 | 603 | 603 | 603 | R=82 |
| | (1,013,762) | (43) | (47) | (50) | (49) | | | U= 2 |
| River-clay soils | 193 | 551 | 603 | 635 | 631 | 596 | 605 | R=90 |
| | (723,928) | (43) | (40) | (46) | (41) | | | U= 3 |

Rural municipalities : municipalities with <5,000 inhabitants.
Urban ,, ,, ,, >20,000 ,,

For explanation of cover-sand soils see Table II.

### *Reliability of Mortality Data in the Netherlands.*

Holland is a very favourable area for detailed geographical-pathological studies.

(1) Registration of deaths is accurate.

(2) Secrecy of death certificates is assured, so there is no hesitation of the part of the physicians to indicate the real cause of death.
(3) The mortality data are collected by the Netherlands Central Bureau of Statistics, a highly competent institution.
(4) Except in the largest cities, the population does not migrate very much. In the smaller agricultural municipalities migration can practically be neglected.
(5) Physicians from the medical state universities in the Netherlands, with similar educational facilities, are spread all over the country and students from one particular university do not concentrate after graduation in particular areas.
(6) All over the country well-equipped hospitals occur with modern X-ray and other facilities.
(7) In the smaller municipalities the population lives on the local food growing in the neighbourhood and local animals are used for meat consumption.

*Factors Obscuring the Soil-Cancer Relationship.*

As many important factors affect the cancer death rate it is evident that strong variations in those factors may obscure the soil-cancer relationship. For this reason a perfect correlation cannot be expected particularly if different decades are compared. Some of the main disturbing factors can be summarized as follows :

(1) Differences in intensity of other factors affecting the stomach-cancer mortality differing in the various soil groups and for different decades.
(2) Increase of the carcinoma death rate (for various reasons) during the period 1900–1940, the rate varying for the different municipalities on the various soil units.
(3) Changing character and different growing speed of the various municipalities changing the social, professional and other conditions.
(4) Consumption of vegetable food grown on soils different from those surrounding the municipalities.
(5) Local differences in acidity, moisture content in the same type of soil which may change considerably the local chemical balance of soils ; *e.g.* a soil normally provided with magnesium may act locally as a magnesium deficient soil if the soil is very acidic as a result of seasonal flooding by rivers. Poor drainage and water logging often mobilizes the fixed phosphorus in soil and increases the availability to plants. It also affects the amount of organic compounds in soil and may cause certain trace element deficiencies.
(6) Differences in climate during different decades: *e.g.* in the province of Zeeland manganese deficiency diseases occur in plants after very long dry periods, especially on soils rich in humus, the influence being different for different types of soil and during different decades.
(7) Differences in exploitation of soil during different decades : *e.g.* considerable changes occurred in the chemical balance of soil in the Netherlands during the last 80 years because of changes in drainage, heavy manuring, ploughing up of grassland, reclamation of peat and heath areas, etc.

*Cancer of the Stomach in the Netherlands in Relation to Soil.*

For the period 1946–1952 two calculations were carried out :

(1) The total number of deaths from cancer of the stomach in men above the age of 50 in municipalities located on a particular soil unit, were related to the population of 10,000 men above the age of 50 and the percentage of "plus" municipalities was determined (see above). Except for cover sands, the same succession was found as indicated in Table I (particularly the decade 1930–1939): reclaimed peat soils: 80 per cent; peat soils: 61 per cent; sea clay soils : 64 per cent; sandy soils: (all types together): 58 per cent; river clay soils: 50 per cent; cover sands: 68 per cent.

(2) A similar calculation was carried out for each of the 5 year age groups above 50 in order to exclude any possible influence of differences in age group structure. The results are compiled in Table II.

TABLE II.—*Stomach Cancer Mortality on Various Soil Units.*

| Soil unit. | Number of deaths from cancer of the stomach per 10,000 of the male population living on the various soil units (during the 7-year period 1946–1952) in the following age groups | | | | | | | Total number of deaths in men, above 50 years, from cancer of the stomach, during the 7-year period 1946–1952. |
|---|---|---|---|---|---|---|---|---|
| | 50–54 (1895) | 55–59 (1890) | 60–64 (1885) | 65–69 (1880) | 70–74 (1875) | 75–79 (1870) | 80+ (1865) | |
| Reclaimed peat soils | 55 | 82 | 137 | 189 | 359 | 482 | 648 | 255 |
| Sea-clay soils | 36 | 72 | 121 | 202 | 318 | 470 | 643 | 2969 |
| Peat soils | 44 | 83 | 107 | 258 | 553 | 439 | 600 | 433 |
| Cover sands | 45 | 69 | 121 | 198 | 357 | 465 | 559 | 2013 |
| Sandy soils (all types) | 39 | 67 | 110 | 186 | 325 | 436 | 553 | 4991 |
| Ordinary sandy soils | 33 | 65 | 99 | 176 | 302 | 407 | 522 | 2190 |
| River-clay soils | 32 | 51 | 104 | 167 | 251 | 414 | 467 | 1360 |
| Average for the country | 35 | 64 | 109 | 188 | 304 | 443 | 567 | Total for country 14,072 |

(1) Numbers between brackets in the age group columns indicate the approximate year of birth of this particular age group.
(2) Cover sands : rather fine, well sorted sands with grain sizes particularly between 105 and 210 mμ, deposited by wind action at the end of the Würmglacial period.
(3) Ordinary sandy soils : sandy soils usually as a result of heath reclamation.
(4) The total of 14,072 includes deaths on soil units not mentioned in Table II because the total population living on those soil units is very small.

Table II warrants the following conclusions :

(1) In all age groups the river clay soils have a lower average stomach cancer mortality than the seaclay soils. The same relationship holds for the sandy soils (all types), peat soils and reclaimed peat soils as compared with the riverclay soils.

(2) The ordinary sandy soils have higher average stomach cancer mortality than the river-clay soils in 5 of the 7 age groups ; the reclaimed peat soils, peat soils and sea-clay soils have higher rates than the ordinary sandy soils in all age groups.

(3) The cover sands have higher average stomach cancer mortality rates than the ordinary sandy soils in all age groups.

(4) A comparison between the other groups indicates that in 4 to 6 of the 7 age groups the succession in stomach cancer rates remains the same.

In order to establish whether, despite these irregularities, the correlations can be considered to be significant, a mathematical study was carried out by Dr. C. A. G. Nass, Head of the Department of Medical Statistics of the Netherlands Institute of Preventive Medicine. I wish to express my gratitude for his assistance in this matter. The result of the analysis is compiled in Appendix No. 2. The final conclusion can be summarized as follows : a true association between stomach cancer mortality and types of soil is highly probable on the basis of a " variance test " using ranked mortalities. A mathematical " sign " test supports this statement.

(5*a*) In all age groups (except one, in case of the sandy soils) the river-clay soils and ordinary sandy soils have average stomach cancer mortalities below the average of the country and can be classified as " minus soils " For the oldest age groups it is true for all sandy soils (also the cover sands).

(*b*) seaclay, peat and reclaimed peat soils are " plus soils " in all age groups.

(6) The increase in stomach cancer mortality with increasing age is very great after the age of 65. The ratios between the stomach cancer mortality in the age group 80 and older and the group 50–54 is highest for the sea clay soils (17·8), followed by river-clay (14·6), sandy soil (all types : 14·2), peat soil (13·6), reclaimed peat soil (11·8). The same succession is found for the ratio of the age group 75–79 and 50–54. In other words, except for the seaclay soils, one has the impression that the increase in the oldest age groups is inverse with the average mortality, *i.e.* greatest increase in soils with lowest average stomach cancer mortality.

*Possible Causes of the Soil-Stomach Cancer Relationship.*

We have seen that differences in age group or sex could not explain the observed relationship. Still there are a number of factors which could be responsible for such a relationship : differences in racial and genetic factors (and blood groups), differences in type, character and growing speed of municipalities on the various soil groups.

The geographical distribution maps of cancer of the stomach were discussed with experts of the Department of Anthropogenetics, of the Netherlands Institute of Preventive Medicine. No obvious correlations were found between soil units and racial or genetic groups in the Netherlands. Blood group data in the Netherlands are insufficient for such a comparative study. In view of the observation of Aird and Bentall (1953) that cancer of the stomach seems to be more frequent amongst people of Blood Group A and particularly rare in Group O, it is worth while to study in future this possible cause of the observed correlation.

Differences in type and character of municipalities cannot explain the observed relationship because, as indicated in Table I, sea-clay soils and river clay soils have almost the same percentage of rural municipalities (90 per cent). In fact we do not know of any factor which could give a reasonable explanation of the observed correlation. Therefore we are inclined to believe that the relationship, at least partly, is a true causal relationship. We do not like to enter into any speculative hypotheses at the present stage of research. However, we should like to point out only that if certain trace element distributions in soil, drinking water and food would affect the origin and (or) development of cancer of the stomach, for example by disturbing the enzyme balance in the body cells (as suggested by the studies of van Everdingen (1952) and others) or in counteracting the glycolitic and other processes in cells (as suggested by the studies of Hecht and Eichholtz (1929) and others), the soil-cancer relationship could be more easily understood.* In Table III a summary is given of the known distribution of a number of trace elements in the Netherlands. Differences in precipitation in different years, differences in drainage, acidity, lime, potassium, phosphate and iron content, etc., may lower or increase the expected available quantities as indicated in Table III. It is well known that several cattle diseases in the Netherlands and in other countries are closely related to soils with certain trace element deficiencies (or surpluses as in the case of Mo). Therefore it seems reasonable to assume that also " man " may be affected by certain trace element distributions. For iodine and fluorine it is even a generally accepted fact. If this would be true for other elements too, it seems possible that the observed soil-cancer relationship may prove to be, at least partly, a soil-trace element-cancer relationship. The second author, together with Dr. Lehr (Director of the Plant Nutrition Research Laboratory at Wageningen), is planning to carry out further research on this subject in the near future.

TABLE III.—*Distribution of Trace Elements in Soils in the Netherlands.*

| Copper. | Cobalt. | Boron. |
|---|---|---|
| Little known about actual Cu contents of soils in the Netherlands<br>1. Sea-clay<br>River-clay<br>2. Reclaimed heath-soils (ordinary sandy soils) | 1. Peat soils<br>Sea-clay<br>River-clay<br>2. Sandy soils | 1. Sea-clay<br>2. River-clay<br>3. Reclaimed peat soils<br>4. Sandy soils<br>(*a*) If rich in organic matter often richer in Boron.<br>(*b*) High acid and high lime content increase Boron deficiency |

| Iodine. | Magnesium. | Manganese. | Molybdenum. |
|---|---|---|---|
| 1. Sea-clay<br>2. Peat soils<br>3. River-clay<br>4. Sandy soils | 1. Sea-clay<br>2. Peat soils<br>3. River-clay<br>4. Sandy soils<br>High acid and potassium content decreases available amount of MgO (*e.g.* flooded areas) | 1. River-clay<br>2. Peat soils<br>3. Sea-clay<br>4. Sandy soils<br>Reclaimed Peat soils<br>Low acid, high P and Fe, high $CaCO_3$ and humus content lowers available amount of Mn | Surplus of Mo causes disease.<br>1. Light sea-clay (Wieringermeer)<br>2. Peat soils (Z.H.)<br>3. Frisian Sea-clay<br>4. Sandy soils<br>Poorly drainage, alcaline soils and high Co content increases available amount of Mo |

Numbers 1–4 indicate that the trace element content per kg. soil decreases from Group 1 to 4 (trace element deficiency).

* According to Hecht and Eichholtz (1929) particularly copper, but also zinc and bismuth seem to affect glycolitic processes in tumour cells.

### Appendix 1 (*a*).

### *Mathematical Study of the Mortality Data of Table I.*

(By the Department of Medical Statistics of the Netherlands Institute of Preventive Medicine at Leiden, Holland.)

For each decade the mortality figures of Table I can be ranked as indicated in Table IV.

### Table IV.

| Soil unit. | Ranked mortality figures. 1900–1909. | 1910–1919. | 1920–1929. | 1930–1939. | Totals. |
|---|---|---|---|---|---|
| Reclaimed peat soils | 1 | 1 | 3 | 2 | 7 |
| Peat soils | 2 | 2 | 1·5 | 3 | 8·5 |
| Sea-clay soils | 3 | 3 | 1·5 | 1 | 8·5 |
| Sandy soils (all types) | 4 | 4 | 4 | 4 | 16 |
| River-clay soils | 5 | 5 | 5 | 5 | 20 |
| Totals | 15 | 15 | 15 | 15 | 60 |

*Analysis of variance :*

| Source. | Sums of squares. | Degree of freedom. | Mean squares. |
|---|---|---|---|
| Period | — | 3 | — |
| Types of soil | 32·375 | 4 | 8·094 |
| Error | 7·125 | 12 | 0·594 |
| | 39·500 | 19 | |

$F = 8{\cdot}094/0{\cdot}594 = 13{\cdot}6$

With 4 and 12 degrees of freedom the 0·1 per cent singificance level of $F$ is 9·63, so the association between cancer mortality and types of soil is significant.

### Appendix 1 (*b*).

### *Mathematical Study of the Percentages " Plus " Municipalities of Table I.*

(By the Department of Medical Statistics of the Netherlands Institute of Preventive Medicine at Leiden, Holland.)

For each decade the percentages " plus " municipalities can be ranked as indicated in Table V and a " variance analysis " can be applied on these figures.

### Table V.

| Soil unit. | Ranked percentages " plus " municipalities. 1900–1909. | 1910–1919. | 1920–1929. | 1930–1939. | Totals. |
|---|---|---|---|---|---|
| Reclaimed peat soils | 1 | 1 | 1 | 1 | 4 |
| Peat soils | 2 | 2 | 2 | 3 | 9 |
| Sea-clay soils | 3 | 3 | 3·5 | 2 | 11·5 |
| Sandy soils (all types) | 4 | 4 | 3·5 | 4 | 15·5 |
| River-clay soils | 5 | 5 | 5 | 5 | 20 |
| Totals | 15 | 15 | 15 | 15 | 60 |

*Analysis of variance :*

| Source. | Sums of squares. | Degree of freedom. | Mean squares. |
|---|---|---|---|
| Period | — | 3 | — |
| Types of soil | 37·375 | 4 | 9·344 |
| Error | 2·125 | 12 | 0·177 |
| | 39·500 | 19 | |

$F = 9{\cdot}344/0{\cdot}177 = 52{\cdot}8.$

With 4 and 12 degrees of freedom the 0·1 per cent significance level of $F$ is only 9·63, so the association between percentages "plus" cancer municipalities and types of soil is highly significant.

### APPENDIX 2.

### *Mathematical Study of the Mortality Data of Table No. 2.*

(By the Department of Medical Statistics of the Netherlands Institute of Preventive Medicine at Leiden, Holland.)

The variance of the mortality figures clearly depends on the age groups. In the age group 50–54 it is apparently smaller than in the age group 80 and more. This fact prevents the immediate application of a "two-ways analysis of variance." The difficulty can be removed by ranking the mortality figures for each age group separately a indicated in Table VI.

TABLE VI.

| Soil unit. | Ranked mortality figures according to age groups. 50–54. | 55–59. | 60–64. | 65–69. | 70–74. | 75–79. | 80 and more. | Totals. |
|---|---|---|---|---|---|---|---|---|
| Reclaimed peat soils | 1 | 2 | 1 | 4 | 1 | 1 | 1 | 11 |
| Sea-clay soils | 4 | 3 | 2 | 2 | 4 | 2 | 2 | 19 |
| Peat soils | 3 | 1 | 4 | 1 | 3 | 4 | 3 | 19 |
| Cover sands | 2 | 4 | 3 | 3 | 2 | 3 | 4 | 21 |
| Ordinary sandy soils | 5 | 5 | 6 | 5 | 5 | 6 | 5 | 37 |
| River-clay soils | 6 | 6 | 5 | 6 | 6 | 5 | 6 | 40 |
| Totals | 21 | 21 | 21 | 21 | 21 | 21 | 21 | 147 |

*Note :* The group Sandy soils (all types together) was left out purposely because the mortality figures consist mainly of the group Cover sands and ordinary sands.

*Analysis of variance :*

| Source. | Sums of squares. | Degree of freedom. | Mean squares. |
|---|---|---|---|
| Age groups | — | 6 | — |
| Types of soil | 93·1 | 5 | 18·6 |
| Error | 29·4 | 30 | 0·980 |
| | 122·5 | 41 | |

$F = 18{\cdot}6/0{\cdot}980 = 18{\cdot}98.$

With 5 and 30 degrees of freedom the 0·1 per cent significance level of $F$ is only 5·12, so a true association between stomach cancer mortality and types of soil is highly probable.

Pairwise comparison of the types of soil is possible with the "sign test". With only 7 pairs, significance at the 5 per cent level requires that all mortality figures of one type of soil are higher than the corresponding mortality figure of another type of soil.

The figures of Table II indicate that :

(1) the mortality figures of riverclay and ordinary sandy soils are significantly lower than those of other types of soil ;

(2) the mortality figures of cover sands are significantly lower than those of the reclaimed peat soils.

### REFERENCES.

AIRD, I. AND BENTALL, H. H.—(1953) *Brit. med. J.* i, 799.
DAVIES, R. I. AND WYNNE GRIFFITH, G.—(1954) *Brit. J. Cancer*, **8**, 56.
DIEHL, J. C. AND TROMP, S. W.—(1954) Foundation for the Study of Psycho-Physics. First report on the geographical and geological Distribution of Carcinoma in the Netherlands, Oegstgeest, Holland.—(1954) *Experientia*, **10**, 12, 510.—(1954) 'Foundation for the Study of Psycho-Physics.' 2nd Annex to First Report.
VAN EVERDINGEN, W. A. G.—(1952) *Ned. Tijdschr. Verlosk.* 5.
HECHT, G. AND EICHHOLTZ, F.—(1929) *Biochem. Z.*, **206**, 282.
WYNNE GRIFFITH, G. AND DAVIES, R. I.—(1954) *Brit. J. Cancer*, **8**, 594.